# The Role of Fractional Radiofrequency in Long-term Acne Remission and Reduction of Acne Scar Load

**DOI:** 10.1093/asj/sjae150

**Published:** 2025-01-16

**Authors:** Fadi Hamadani, Neil M Vranis

## Abstract

**Background:**

Acne is an inflammatory skin disease afflicting the majority of the world's population at some point in their lifetime, and is seen to be chronic in about 50% of cases. Acne leads to significant social withdrawal, depression, and disfiguring scars in many cases. Available treatments are characterized by high rates of relapse, dangerous side effects, and social stigma, which often leads to poor patient compliance and treatment failure.

**Objectives:**

The aim of this article was to discuss and share the authors’ experiences utilizing fractional radiofrequency (RF) (Morpheus8; InMode Ltd., Lake Forest, CA) in the treatment of both active acne and acne scars.

**Methods:**

A retrospective review was conducted comparing 3 treatment modalities. In total, 356 patients received acne scar treatments. The cohort comprised a high-dose isotretinoin topical therapy series (n = 128, 36%), a 6-session ablative laser series (n = 89, 25%), and a 3-session fractional RF microneedling series (n = 139, 44).

**Results:**

Of the patients with extended 3-year follow-up, the relapse rates were: isotretinoin group, 36 of 54 (67%); laser group, 12 of 16 (75%), and fractional RF microneedling group, 7 of 29 (24%).

**Conclusions:**

In treating older acne scars, fractional RF microneedling technology has served as an effective tool to tighten skin and fill in atrophic scars when used in conjunction with other techniques. This technology is very effective and very safe for treating all skin types with acne and acne scars.

Acne is the most common skin disease globally, with over 85% of the world's population having been afflicted at least once in their lifetime.^1^ Increasingly, acne is being recognized as a chronic disease, as it waxes and wanes in over 50% of sufferers and often continues into adulthood.^2^ The complexity of the disease, its well-documented social impact, and the lack of consistent and safe treatments have led an ongoing search for new therapies.^3,4^

The pathogenesis of acne is a complex interplay of genetic predisposition, hormonal imbalances, and environmental triggers that leads to hyperkeratinization of the pilosebaceous unit, follicular desquamation, colonization by *Cutibacterium acnes* (formerly *Propionibacterium acnes*), and an inflammatory cascade of cytokines.^5-7^ There is mounting evidence that acne has a strong autoimmune component in over 50% of cases.^8^

The social impact of acne is also well documented. Many patients suffer from disfiguring scars that lead to social withdrawal and depression, often because they fail to seek early treatment.^9^ The stigma surrounding many acne treatments, together with an unfavorable side-effect profile, has led many patients to self-medicate or to receive ineffective therapies.^10^ Traditional therapies have included oral and topical retinoids and antibiotics, hormonal medications, and a large selection of topical keratolytics.^11,12^ There has been a drive to find new and effective therapies for both active acne and acne scars because of the huge market potential.^13^

Some of the alternative therapies for acne that have shown promise include lasers, fractional techniques, chemical peels, and light therapies,^14,15^ but none have been consistent in their results and there are high levels of relapse. Microneedling, a technique that was developed in the 1990s, utilizes fractional and controlled-depth penetration of the skin with different types of microneedles. Microneedling has been shown to induce a wound-healing cascade of growth factors and cytokines that can improve acne scars, wrinkles, and striae,^16^ but has a very limited number of applications in treating active acne.

The Morpheus8 (InMode Ltd, Lake Forest, CA) is a fractional radiofrequency (RF) system that delivers thermal energy to variable depths of the skin through 24 parylene-coated gold-plated pins^17^ (Figure 1). Fractional RF microneedling has a well-documented effect on improving the thickness of the reticular and papillary dermis connective tissue and fibers of the skin by inducing a cascade of growth factors, both in the face and body.^17,18^ The distal part of the tip, which is uncoated, creates a compact zone of ablation surrounded by 2 expanded zones of necrotic and subnecrotic nonspecific heating.^17^ This leads to a significant tightening of the skin in addition to a noticeable improvement in skin quality via subacute (up to 3 months) neocollagenesis. The treatment acne and acne scars with fractional RF microneedling devices has not previously been described.

**Figure 1.**
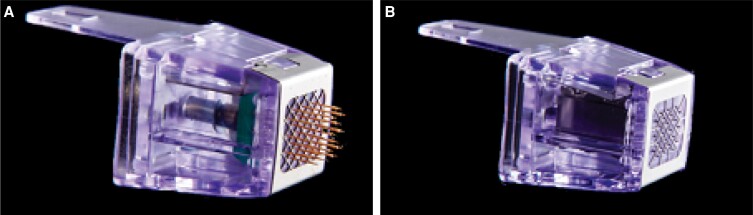
Fractional radiofrequency tips. (A) Morpheus8. (B) Morpheus8 T Resurfacing (InMode Ltd, Lake Forest, CA).

The purpose of this Supplement is to introduce the utility of applying fractional RF microneedling for the treatment of acne in all its forms, with the goal of providing long-term remission, considered as a relapse rate of less than 15% in a 5-year period, and effective early scar remodeling. Furthermore, specific protocols for late acne scar remodeling that utilize fractional RF in combination with other modalities will also be discussed.

## DIFFERENCES BETWEEN FRACTIONAL RADIOFREQUENCY TECHNOLOGIES

There are different forms of RF energy with medical applications. Most available systems are bipolar RF, meaning that the energy alternates between positively and negatively charged electrodes situated close together within the system.^19^ Most fractional devices on the market today consist of a row of positively charged electrodes positioned parallel to a row of negatively charged ones, so that the RF arc travels a very small distance between the pairs of tips. This configuration results in a very small and superficial zone of ablation, and the induced wound-healing cascade is not very different than standard needling without RF.^20^

The Morpheus8 bipolar fractional RF microneedling system is designed differently and offers noticeable advantages. The electrode pins are all positively/negatively charged, with the grid surrounding each pin located at the interface of the tip near the skin^17,20^ having the opposite charge. As such, the RF loop is large and induces a bigger and deeper zone of ablation or coagulation that leads to a large quantity of released cytokines and growth factors. Fractional RF treatment spares the epidermis, except for the mechanical injury to the skin, and is considered to be significantly safer in darker skin types.^21^

## TREATMENT PROCEDURE FOR ACTIVE ACNE AND EARLY ACNE SCARS

### Diagnosis and Pretreatment

Active acne is identified clinically through the identification of well-known lesions on the face such as closed comedones (blackheads), open comedones (whiteheads), and inflammatory papules and pustules. Often, the lesions lie on a background of erythema with varying degrees of scars depending on the severity of the acne.^22^ The majority of acne scars fall into the following categories: icepick, boxcar, and rolling.

A detailed family history and physical exam are undertaken with special attention paid to recent use of isotretinoin. Although several studies have demonstrated the safety of both ablative and nonablative energy-based devices for patients on isotretinoin, in darker skin types we opt to reduce the energy by 20%. Contraindications to treatment include high doses of corticosteroids, immunocompromised states, active infections other than acne, pregnancy, and unrealistic expectations.

### Skin Preparation and Anesthesia

Before the treatment, the skin is cleaned and exfoliated with a keratolytic toner. At high energies, fractional RF microneedling can be quite painful and it is important to provide safe and appropriate analgesia. Options for anesthesia include application of topical numbing creams, nerve blocks, or local anesthesia. One helpful option for anesthesia comes from administering a modified tumescent solution that consists of 60 mL normal saline, 400 mg lidocaine, and 0.3 mg epinephrine. This is then administered via a special chamber that connects to the syringe and allows the tumescent to flow through multiple small needles. When sufficient anesthesia is achieved, the face is cleaned with alcohol wipes.

### Treatment

During the treatment both the standard fractional RF microneedling tip and the “resurfacing tip” were used. Both tips consist of 24 pins, but the former is coated (except the distal 0.5 mm) and can reach depths of 1 to 4 mm, whereas the latter is noncoated and fixed at a depth of 0.5 mm. Table 1 provides an outline of the settings used based on Fitzpatrick skin type. The treatment starts with passes at the deeper layers first and continues superficially. Depending on skin thickness, a depth of 3 mm or 2 mm is chosen to commence the treatment. Once the tip is placed firmly against the skin, a foot pedal is used to activate the device and deliver the pulses of energy. In general, at depths of more than 2 mm, the device is set to “fixed mode,” which enables it to deliver several pulses of RF energy through the pins continuously as long as the foot pedal is engaged. The system delivers an acoustic signal to indicate the delivery of each pulse, and the tip is activated to deliver double-stacked pulses at the predetermined depth. Once the double-stacked pulses are delivered, the handpiece is moved over the skin such that it overlaps 50% with the previous treatment area. A set of double-stacked pulses is delivered to each site. Once the treatment zone has received 1 pass of stacked pulses, a second pass that overlays the previous treatment is conducted. This ensures that the treatment zone has received 2 passes of 2 stacks each. Following this, the more superficial depth is started, to ensure full coverage of the treatment zones. At depths of 2 mm or less, the “cycle mode” is preferred because this reduces the risk of skin surface overexposure to heat. This mode allows for the pins to insert and deliver the preset amount of RF energy before retracting, only to be reintroduced for the second pulse. Once the treatment is completed, a moisturizing lotion is applied.

**Table 1.** Morpheus8 Treatment Parameters Based on Fitzpatrick Skin Type

Condition	Treatment area	Depth (mm)	RF energy level	Mode
Active acne, skin type I-IV	Forehead	1 mm	25	Cycle
	Cheeks/jaw	2 and 3 mm	30-45	Fixed
	Neck	2 mm	30-40	Cycle
Active acne, skin type V-VI	Forehead	1 mm	20	Cycle
	Cheeks/jaw	2 and 3 mm	25-35	Fixed
	Neck	2 mm	25-35	Cycle
Acne scars	Forehead	1 mm	25	Cycle
	Cheeks/jaw	2 and/or 3 mm	30-40	Fixed

### Posttreatment

Patients are instructed to avoid sun exposure and to moisturize the treated skin multiple times a day. Patients on topical retinols are instructed to avoid using them for at least 1 week. Patients on oral isotretinoin are instructed to continue taking their prescribed medications. Sun protection is emphasized, as is avoiding exposure to any other direct heat sources for at least 30 days. Patients are informed of expected side effects, including swelling, erythema, crusting, and itchiness and are informed that these may last for several days.

## TREATMENT PROCEDURE FOR OLD ACNE SCARS

### Diagnosis and Pretreatment

Old acne scars are identified according to their clinical morphology, and classified as ice-pick, boxcar, or rolling scars^1,23^ (Table 2). This nomenclature provides a general outline of scar morphology and is meant to serve as a guide to scar depth. We have found it to be more useful to think of each subtype as existing on a spectrum, starting with pores on one end, through to pits and then ice-picks on the other end. In the case of boxcar scars, they may present as narrow or wide, deep or shallow. Rolling scars have a classical peau d’orange appearance and always improve when light traction is applied to the skin. Although classically associated with rolling scars, it is our experience that all acne scars have a degree of tethering due to fibrotic bands between the skin and subcutaneous tissue, often reaching the muscle.

**Table 2.** Main Acne Scar Subtypes^1,23^

Subtype	Description
Ice-pick scars	Narrow and deep scars
Boxcar scars	Broad or narrow, deep or shallow scars with sharp edges
Rolling scars	Broad scars with a sunken appearance and tethering that improve on skin traction

It is important to note the patient's skin type, and to determine if active acne is still present. Patients with active acne are encouraged to treat the active lesions with fractional RF microneedling or other modalities before commencing with scar revision. Darker-skinned patients are encouraged to condition the skin with topical retinol or tyrosinase inhibitors for at least 3 to 4 weeks prior to commencing treatment with fractional RF microneedling. Skin preparation and anesthesia are carried out as described above.

### Treatment

#### Ice-pick Scars

Fractional RF microneedling can remodel ice-pick scars because the pins are able to penetrate the skin at varying depths. In our experience, performing a trichloroacetic acid (TCA) or phenol chemical reconstitution of skin scars (CROSS) or even a punch excision of the scar with a disposable 1-mm skin punch device works in synergy with fractional RF microneedling to produce outstanding results for this scar type.^24,25^ A depth of 2 mm in “cycle mode,” with an energy level ranging from 35 to 45, is selected. RF delivery of a single pulse with 75% overlap and 2 or 3 passes is applied.

#### Boxcar Scars

These scars respond extremely well to fractional RF microneedling and rarely need any adjunctive therapy. Both the depth of the scar and the consistency of the skin improve notably. The same settings and treatment protocol as for ice-pick scars are used.

#### Rolling Scars

Almost all atrophic acne scars, by definition, have a certain degree of tethering.^26^ The most important adjunctive procedure in our experience for this scar type is tumescent subcision.^26,27^ The same anesthesia described above is delivered through a 22G, 70-mm cannula until the skin is turgid. A 2-cm region outside of the mapped scars is anesthetized. Utilizing the same cannula, or a larger caliber one (up to 16G can be used for very fibrotic tissue), repeated gentle strokes parallel to the skin surface are made to break through the bands of the fibrosis.^28^ The key is to be firm and slow. Multiple strokes, in parallel passes, are made until a full release of the tethering is accomplished. The endpoint is identified as when the ease of the passage of the cannula increases. An audible sound of the bands breaking is often heard. Fractional RF microneedling is then performed, with the tumescent providing the necessary anesthesia. For rolling scars, the energies are often the same as those for ice-pick and boxcar scars. The option to inject an agent for collagen boosting is also available, although we prefer to carry out this step at a later time, at least 2 weeks after the simultaneous subcision and fractional RF microneedling treatment session.^27,29^

## ANCILLARY TECHNIQUE UTILIZING ACCUTITE FOR SUBCISION OF SCARS

**Figure 2.**
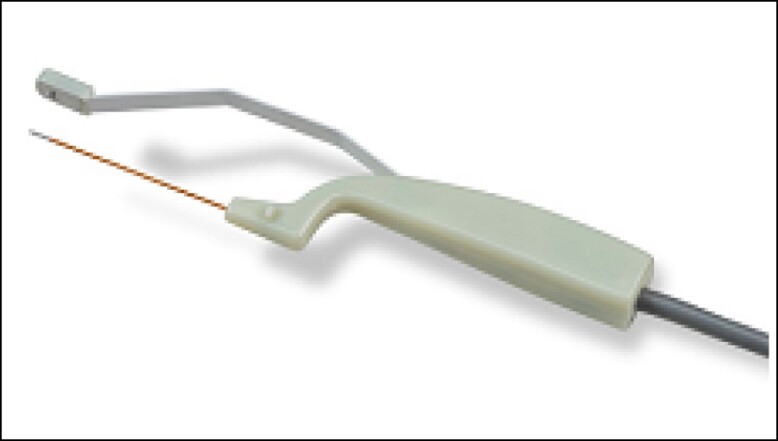
AccuTite handpiece (InMode Ltd, Lake Forest, CA).

The AccuTite handpiece (InMode Ltd, Lake Forest, CA) (Figure 2) is a smaller version of the FaceTite probe, which has been described previously.^17,18,30^ Both AccuTite and FaceTite handpieces (InMode Ltd, Lake Forest, CA) are minimally invasive bipolar RF devices that produce deep tissue heating to tighten the overlying fibroseptal network. An internal positively charged electrode and an external negatively charged electrode complete the circuit. The AccuTite handpiece consists of a 6-cm cannula, 0.9 mm in diameter, and is used to treat more sensitive areas of the face such as the nasolabial folds and periorbital area.

**Figure 3.**
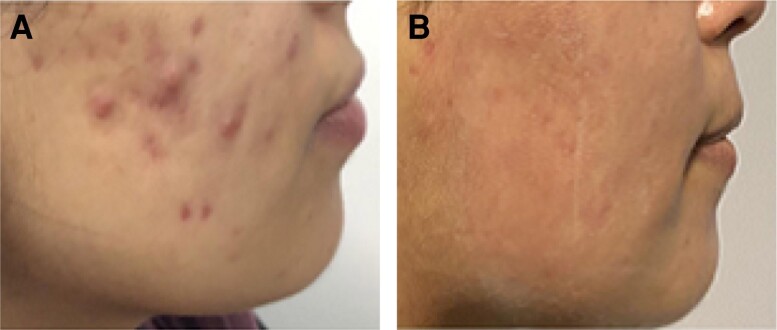
Active acne and early acne scar resolution after a treatment protocol with low-dose isotretinoin (10 mg, 3× per week, 6 months) and 3 sessions of fractional radiofrequency microneedling in a 27-year-old female. (A) Pretreatment. (B) Six months posttreatment.

We have developed a new application for this powerful handpiece, using the internal electrode as an internal cannula for subcision. Once the scar bands are mechanically released, RF is delivered at very conservative temperatures: 50°C internal and 35°C external. When treating acne scars with this technique, it is not necessary to reach the cut-off temperatures, but rather to deliver a few passes in the subcised zone. We have found this to be sufficient for remodeling the skin. Figure 3 demonstrates a typical result achieved at 6 months follow-up visit after the last treatment. The treatment protocol included 2 sessions of fractional RF microneedling treatments for active acne, followed by 3 sessions of minimally invasive bipolar RF with subcisions as described above. In the final session, the patient was administered a collagen booster (calcium hydroxylapatite, diluted 1:1 with lidocaine 2%; Radiesse, Merz Pharma GmbH & Co., Germany), immediately after this treatment session.

## EXAMPLES OF CLINICAL APPLICATIONS OF FRACTIONAL RADIOFREQUENCY FOR VARIOUS ACNE PRESENTATIONS

### Results of Treatment of Active Acne and Preventing Acne Scars

Many options exist for the treatment of active acne, but high relapse rates and an unfavorable side-effect profile may lead to poor compliance.^31^ Many patients seek alternative therapies that are able to provide longer remission. Many of our patients had undergone previous treatments and had experienced relapse, including treatments with high doses of isotretinoin. Although isotretinoin has been shown to improve and modulate early acne scars, its ability to do so is limited, especially in cases of severe cystic acne.

**Figure 4.**
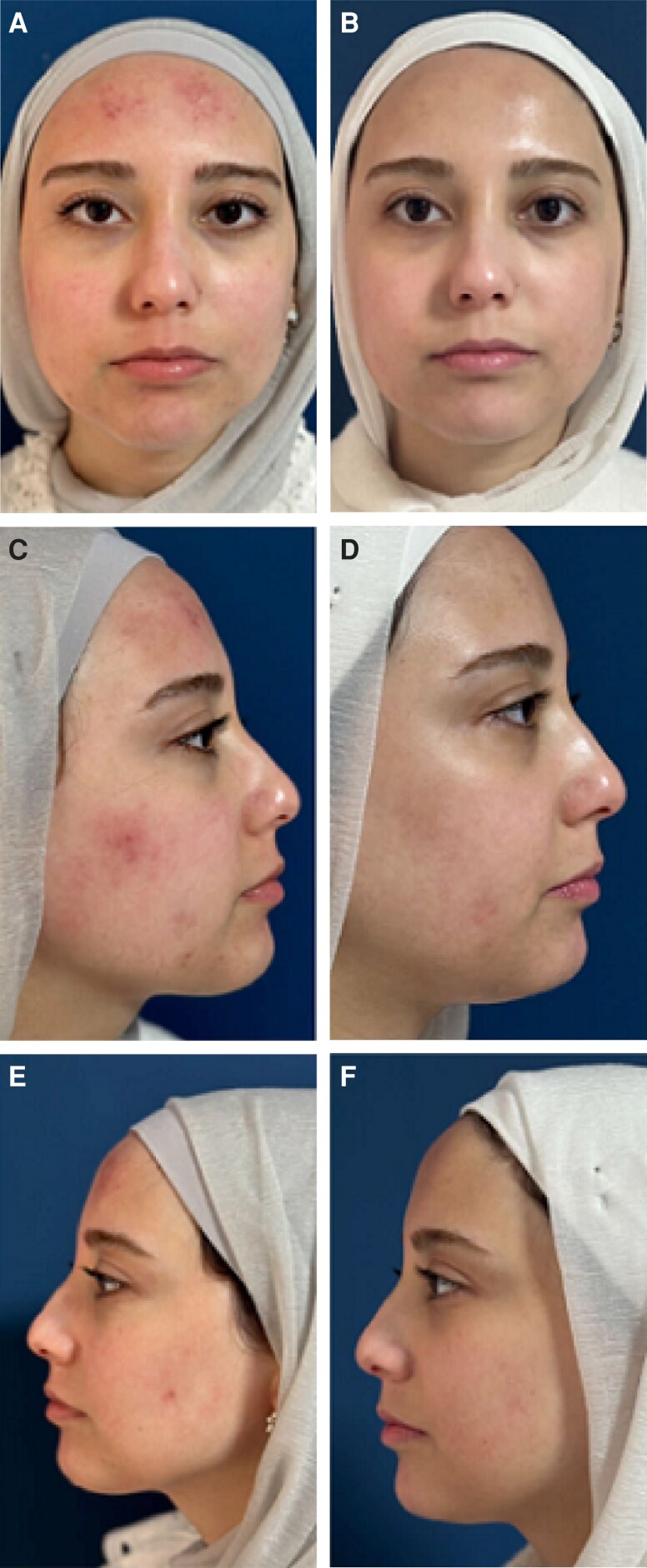
Results of treatment in a patient with acne and rosacea. This 23-year-old female underwent 4 sessions of fractional radiofrequency microneedling spaced 2 months apart. (A, C, E) Pretreatment. (B, D, F) Six months posttreatement.

We have noticed in our practice that fractional RF microneedling has the added benefit of significantly improving early acne scars and acne rosacea with excellent results (Figures 3, 4). Most patients have a complete elimination of early scars which are identified by being red and in the early stages of atrophy. We have also noticed higher remission rates of active acne.

Between February 2018 and October 2021, 356 patients received acne and early acne scar treatments at one of our clinics. Of these patients, 128 (36%) opted for treatment with isotretinoin in high doses and a skincare routine. A group of 89 patients (25%) elected for topical therapy involving 6 sessions with a combination of Er:glass and Nd:YAG lasers. The remaining 139 (44%) patients underwent treatment for their active acne with a skin protocol and fractional RF microneedling. The ancillary skincare protocols for the 3 groups were very similar. All patients were prescribed a keratolytic wash, sun protection, and a topical retinol. Of the 3 groups, a subset of 54 patients of the isotretinoin group, 16 patients of the laser group, and 29 patients of the fractional RF microneedling group have completed a 3-year follow-up since treatment. A large proportion of the patients in the isotretinoin group (36/54, 67%) relapsed within a 3-year period. The laser group had an even higher relapse rate of 75% (12/16 patients). Of the fractional RF microneedling group, 24% experienced relapse (7/29 patients). Satisfaction in this patient group had a mean [standard deviation] of 4.2 [0.4] out of 5. Relapse was defined as patient or practitioner observing worsening of acne or acne scarring after the initial improvement after treatment. There were no adverse outcomes observed—this includes thermal injury to the epidermis, prolonged erythema, or hyper/hypopigmentation.

Although we were unable to objectively quantify the scar burden in all these patients after treatment, the majority of patients treated with fractional RF microneedling were very satisfied with the quality of their skin, whereas patients in the remaining 2 groups would often complain about the scars they were left with after their acne remission.

### Results on Old Acne Scars

Old acne scars present one of the most challenging scar types to treat.^32^ It is difficult to predict response rate and most patients are only able to achieve a modest result.^1^ Darker skin types pose a particular challenge as many of the more aggressive laser options lead to severe pigmentary changes.^33^ Our experience shows that fractional RF treatment for acne scars is a safe and effective option for all skin types when situated within an acne scar revision protocol that combines multiple modalities.

Some of the most effective treatments for the different scar types have been summarized above. We have found fractional RF microneedling to be a powerful adjunct for acne scar revision. Combining RF energy with the other procedures described helps initiate neocollagenesis and dermal remodeling, as well as significant tightening of the skin over time. In addition to lifting the atrophic tissue, neocollagenesis helps to support the soft tissue architecture surrounding the scar, leading to smoothening and textural improvement.

**Figure 5.**
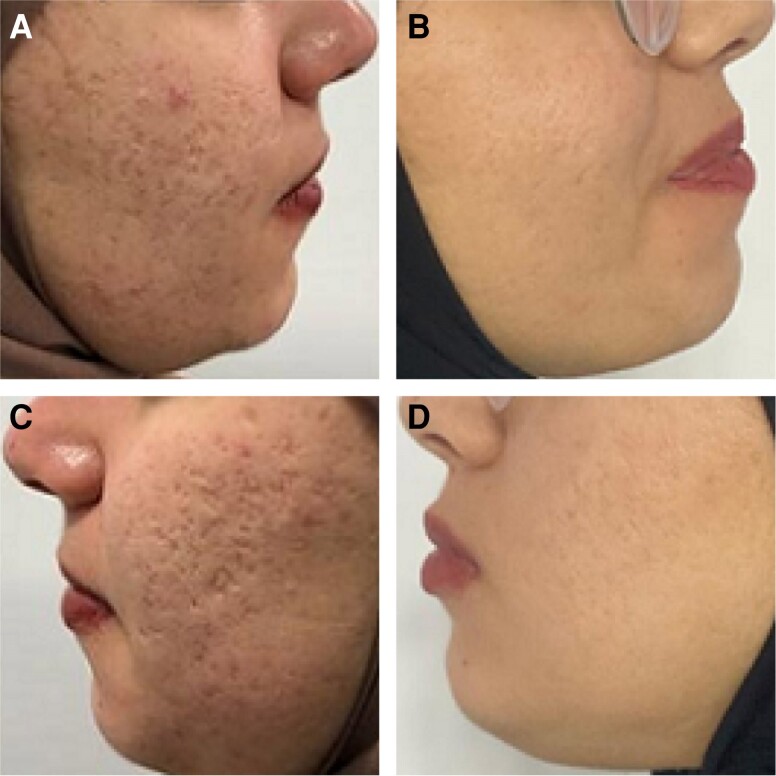
Typical results of acne scar revision protocols utilizing several tools along with fractional radiofrequency microneedling treatment series. In this case, a 25-year-old female underwent punch excision with a 1-mm punch, tumescent subcision, and fractional radiofrequency microneedling. Four sessions were performed every 3 months. (A, C) Pretreatment. (B, D) Nine months after the last session.

The ECCA score (échelle d'évaluation clinique des cicatrices d’acné) is a validated acne scar grading system in use since 2007.^34^ It allows physicians to score acne scars based on number and severity in order to produce an overall score. Between April 2018 and April 2019, 49 patients with average age of 25.4 [5.6] years with varying degrees of old acne scars completed a series of 4 sessions that utilized a combination of treatments together with fractional RF microneedling. After 4 sessions, all patients showed at least a 50% reduction in median ECCA acne scar grading. The average ECCA score for all patients before treatment was 178 [35], but this was reduced after treatment to 132 [17]. Both the number of scars and their depths were significantly reduced, contributing to the overall clinical improvement (Figure 5). Most importantly, there were no significant adverse outcomes for these patients. There were no infections, long-term sequelae of thermal injury, or worsening of scars. Satisfaction in this group of patients had a mean of 3.8 [0.5] out of 5. We did not assess the use of fractional RF microneedling alone for old acne scars.

## DISCUSSION

Available treatments for acne scars often demonstrate mild improvement, but are frequently inconsistent or fail to present a dramatic outcome.^35^ The use of fractional RF has been a powerful addition to our armamentarium of tools used for acne and acne scar treatments. Especially when including fractional RF microneedling in combination with other treatments, we have seen excellent results in scar remodeling, well beyond what has been seen with other treatments. This is most likely due to the powerful energy that fractional RF microneedling can deliver, especially when considering the depth of the RF penetration into the tissue. The advantages that the fractional RF microneedling system has over other fractional and microneedling RF systems on the market may be attributed to the fact that the return electrode is on the surface of the skin, thus allowing the RF current to travel a longer distance, thereby creating a larger and deeper heating zone. Other systems situate the positive and negative electrodes in parallel and the RF current achieved occurs in a small and superficial area between the short tips at the very end of the needles.

The high safety profile achieved with fractional RF microneedling in darker skin types can be attributed to the insulation of most of the length of the pin. In their recent consensus paper looking at energy-based devices for acne scars, Salameh et al showed that noninsulated needles produced less effective results.^36^ One argument is that when the device uses noninsulated needles, the RF spark produced between the positive and negative electrodes occurs along the whole length of the needle. Thus, it produces a more superficial zone of heating, increasing the risk of damage to the skin surface and postinflammatory hyperpigmentation.^36^ However, when the pins are insulated, except for the tip, RF energy is selectively delivered specifically to the target depth. Inadequate treatment of acne scars occurs when treatments are too superficial and do not reach the deep component of ice-pick or boxcar scars.

In 2014, Kaminaka et al were able to show excellent improvements in active acne when they used RF microneedling.^37^ Our experience has been the same, with results from fractional RF microneedling being more consistent than when utilizing lasers or isotretinoin alone. We attribute this to the stronger and deeper heat signal produced by fractional RF microneedling, which in turn leads to a more powerful wound healing response. Furthermore, fractional RF microneedling is known to ablate the sebaceous glands in the skin, and has recently been shown to be an excellent treatment for ectopic sebaceous glands.^38^ Apparently, there is some destruction of the sebaceous glands, resulting in a reduction of sebum overproduction. Histology of adnexal structures in the skin showed higher levels of collagen remodeling than what is seen with laser treatment.^37^ This perhaps explains the observation in our cohort of acne patients that treatment with fractional RF microneedling leads to longer remission rates than treatment with lasers.

The excellent results seen in acne scar revision are probably due to the deeper and more powerful heat signature that fractional RF microneedling devices are able to achieve. Several studies have shown that in addition to significant sebum reduction, RF microneedling is able to upregulate TGFβ, leading to more collagen remodeling, and to downregulate NF-κB and IL-1, associated with improved scar remodeling.^39^ The negative impact of inflammation on scar revision has been well elucidated, and it is apparent that fractional RF microneedling is able to decrease the inflammatory milieu concomitantly with improving collagen remodeling.^37,39^

The current evaluation demonstrates qualitative improvements, as well as a degree of quantitative improvement measured by ECCA scores. Unfortunately, we are currently unable to objectively measure sebum reduction or to histologically confirm the improvements we noted clinically. Limitations of this study also include the retrospective, observational nature of the data. The study was not designed as a randomized, independent, blinded trial, and the patients themselves often chose which treatment protocol to undergo, based on financial limitations and treatment downtimes, introducing a bias. Patients were not asked to keep diaries and there is no independent mechanism whereby we can confirm treatment compliance. Nevertheless, the treatments were offered in the same clinic and by the same physician with similar treatment parameters/settings. The presented data are for the patients who completed the whole treatment plan. The current data also share the longest follow-up period for patients after completing fractional RF microneedling sessions. Most studies have a follow-up of 12 to 18 months,^35^ whereas we have followed our patients for over 3 years. Future directions will include performing isolated fractional RF microneedling treatments in addition to combination treatments and a control group to further elucidate the therapeutic benefits of fractional RF microneedling in patients with acne/acne scarring.

## CONCLUSIONS

Fractional RF microneedling offers the advantage of effectively treating both active acne lesions and the associated early scars, and results in long-term remission. Fractional RF microneedling has proven to be extremely safe in darker-skinned patients, whereas other modalities have many limitations. In addition, applications of fractional RF microneedling can be very effectively expanded to include treatment of old scars.
